# Characterization of a multi-segmented rod-shaped mycovirus within the order *Martellivirales* largely accommodating plant viruses

**DOI:** 10.1016/j.virusres.2025.199591

**Published:** 2025-05-30

**Authors:** Mika Yoshioka, Akihito Fukudome, Yuto Chiba, Daisuke Hagiwara, Syun-ichi Urayama

**Affiliations:** a Laboratory of Fungal Interaction and Molecular Biology (donated by IFO), Department of Life and Environmental Sciences, University of Tsukuba, 1-1-1 Tennodai, Tsukuba, Ibaraki 305-8577, Japan; b Howard Hughes Medical Institute, Department of Biology and Department of Molecular and Cellular Biochemistry, Indiana University, Bloomington, IN, USA; c School of Agriculture, Meiji University, Kawasaki, 214-8571, Japan; d Microbiology Research Center for Sustainability (MiCS), University of Tsukuba, 1-1-1 Tennodai, Tsukuba, Ibaraki 305-8577, Japan

**Keywords:** Mycovirus, RNA virus, Filamentous particle, Plant virus, Viviviridae

## Abstract

The order *Martellivirales* in the *Riboviria* realm includes seven established families. The viruses in this order have a single-stranded positive-sense RNA genome and infect animals, plants, or fungi. In this study, we characterized Aspergillus flavus vivivirus 1 (AfViV1), an RNA virus infecting *Aspergillus flavus* that presumably belongs to the proposed “Viviviridae” family in the *Martellivirales* order. In previous reports, multiple RNA-dependent RNA polymerase (RdRP) sequences related to “Viviviridae” were mainly identified from metatranscriptome data. However, their virological characteristics were not disclosed. Our analysis showed that the AfViV1 virion exhibited a rod-shaped structure with varying lengths and identified the coat protein (CP) encoded by *RNA12* of AfViV1. Using the AfViV1-CP sequence, we detected several potential CP sequences from viruses in the suggested “Viviviridae“ family based on sequence read archive (SRA) data. These data suggest that viruses in this family have similar rod-shaped structures. Interestingly, the AfViV1-CP amino acid sequence was not significantly similar to the known viral CP. However, the predicted structure was similar to rod-shaped viruses in the *Potyviridae* (*Patatavirales*) and *Closteroviridae* (*Martellivirales*) families (and orders). Our analysis describes the first multi-segmented fungal ssRNA virus with rod-shaped particles and expands the morphological diversity of fungal RNA viruses. Additionally, this study highlights the similarities between fungal and plant viruses, suggesting their deep relationships concerning host range, host adaptation, and more.

## Highlights

• First fungal ssRNA virus with multi-segmented rod-shaped particles.
• Coat protein (CP) of AfViV1 detected and used to find related viral CPs.
• Predicted CP structure resembles *Potyviridae* and *Closteroviridae* CPs.
• Expanded morphological diversity in fungal RNA viruses.
• Highlights deep relationships between fungal and plant RNA viruses.

## 1 Introduction

RNA virus diversity has rapidly advanced through sequence identification of a key marker gene encoding RNA-dependent RNA polymerase (RdRP) (Edgar et al., 2022; Neri et al., 2022; Urayama et al., 2024). Consequently, numerous novel RNA virus lineages have been discovered based on RdRP sequence similarity (Hou et al., 2024; Koonin et al., 2024; Wolf et al., 2018). However, the virological properties of these novel RNA virus lineages remain largely uncharacterized. Many of these RNA viruses were identified from RNA samples obtained from multiple organisms, although their hosts were not determined. Consequently, the lack of isolated viral strains has made it difficult to perform detailed virological analyses.

The order *Martellivirales* includes seven established positive-sense, single-stranded (+)ssRNA virus families: *Bromoviridae, Closteroviridae, Endornaviridae, Kitaviridae, Mayoviridae, Togaviridae*, and *Virgaviridae*. Viruses in the *Togaviridae* family are hosted by humans and invertebrates. Viruses in the *Endornaviridae* family are hosted by eukaryotic microorganisms, fungi, and plants, while others are hosted by plants. Within this order, an unclassified lineage has been identified and proposed as "Viviviridae", although its virological characteristics remain unclear. The name is derived from "virga," meaning "rod" in Latin, and is also used in the *Virgaviridae* family, which includes rod-shaped plant viruses such as tobacco mosaic virus (TMV). (Chiapello et al., 2020; Kondo et al., 2022). Most viruses related to "Viviviridae” have been identified from metatranscriptomic data. However, a few viruses related to “Viviviridae” have been detected from isolated fungal strains (Chiapello et al., 2020; Chiba et al., 2021; Degola et al., 2021; Kuroki et al., 2023). These facts suggested that “viviviruses” are fungal RNA viruses and allowed us to reveal the characteristics of “viviviruses”.

In the present study, we focused on Aspergillus flavus vivivirus 1 (AfViV1), an RNA virus infecting *Aspergillus flavus* (Kuroki et al., 2023). Since AfViV1 is suggested to belong to the “Viviviridae” family based on the phylogenetic analysis using the RdRP sequence, we selected AfViV1 for the characterization of "viviviruses". In this study, we characterized the particle structure of AfViV1 and its structural proteins and discuss whether these features are conserved within the proposed viviviruses.

## 2 Materials and methods

### 2.1 Fungal host and virus

Isogenic *Aspergillus* strains that were infected and cured of Aspergillus flavus vivivirus 1 (AfViV1) were previously established (Kuroki et al., 2023). In this study, AfViV1-infected or -cured *Aspergillus flavus* IFM61879 was grown on a potato dextrose agar (PDA) plate at 30°C for 3–5 days or in potato dextrose broth at 30°C for 6 days.

### 2.2 Virus particle purification from mycelia

The fungal mat was cultured in potato dextrose broth for 6 days, harvested by filtration, and stored at −80°C. Approximately 20 g of frozen mycelia were ground in liquid nitrogen and suspended in 200 mL of 0.5 M sodium borate buffer (pH 9.0). The suspension was filtered through a Kimwipe (Crecia, Tokyo, Japan) and the filtrate was clarified by centrifugation at 9,500 × *g* for 15 min at 4°C using a Sovall ST 8R centrifuge (Thermo Fisher Scientific, Germany). The supernatant was ultracentrifuged at 40,000 rpm (V_max_ = 274,111 × *g*) for 1 h at 4°C using an Optima XE-90 Ultracentrifuge (Beckman Coulter, U.S.A.) with a Type 45 Ti rotor. The pellet was resuspended in 1 mL of 0.5 M borate buffer (pH 9.0) and centrifuged at 10,960 × *g* for 1 min at 4°C using an Optima XE-90 Ultracentrifuge with a SW 41 Ti rotor. The supernatant was layered onto a 10–40% sucrose gradient in 0.5 M sodium borate buffer (pH 9.0). Ultracentrifugation was performed at 100,000 × *g* for 2.5 h at 4°C. Fractions 1–10 were collected in 1 mL increments from the top of the tube, and virus-containing fractions were identified using sodium dodecyl sulfate polyacrylamide gel electrophoresis (SDS-PAGE) and quantitative real-time PCR (qRT-PCR). The virus-containing fractions were concentrated and buffer-exchanged using ultrafiltration (Amicon Ultra-0.5 100 kDa Ultracel-PL membrane, Merck Millipore, Japan). The resulting crude virus preparation was layered onto a discontinuous CsCl density gradient and ultracentrifuged at 92,000 × *g* for 3.5 h at 4°C using an Optima XE-90 Ultracentrifuge with a SW 41 Ti rotor. Fractions 1–10 were collected and analyzed as described above. The virus-containing fractions were concentrated to 70 µL via ultrafiltration. Virus particles were stained with Nissin EM stain (Tokyo, Japan) and observed under a transmission electron microscope (H7100 system; Hitachi High Technologies, Tokyo, Japan).

### 2.3 Protein analyses

The stacking and separating gels were prepared using 4.5% and 12.5% of acrylamide. The stacking gel was prepared with 0.5 M Tris-HCl buffer (pH 6.8) containing 0.4% SDS, while the separating gel was prepared with 1.5 M Tris-HCl buffer (pH 8.8) containing 0.4% SDS. The acrylamide solution contained 29.2 g of acrylamide and 0.8 g of *N,N*'-methylenebisacrylamide in 100 mL of distilled water, resulting in a cross-linking percentage of approximately 2.7%. For polymerization, 0.1% ammonium persulfate (APS) and 0.1% *N,N,N*',*N*'-tetramethylethylenediamine (TEMED) were used as the initiator and catalyst, respectively. For each fraction, a 10 µL aliquot was mixed with an equal volume of 2 × sample buffer containing 0.125 M Tris-HCl (pH 6.8), 10% (v/v) 2-mercaptoethanol, 4% (w/v) SDS, 10% (w/v) sucrose, and heat treated at 95°C for 5 min, followed by cooling on ice for one minute. The entire 20 µL of the heat-treated sample was applied to the gel and electrophoresed at 20 mA for approximately 1.5 h. The proteins were fixed by incubating the gel using a fixing solution containing 50% methanol and 10% acetic acid and then stained overnight with Quick-CBB staining solution (FUJIFILM Wako Pure Chemical, Japan). The protein bands presumed to be the coat protein (CP) were analyzed by nano liquid chromatography-tandem mass spectrometry (LC-MS/MS) (JPROteomics, Japan).

### 2.4 Modeling and structural prediction of AfViV1-CP

A custom multiple sequence alignment (MSA) approach was used to predict AfViV1-CP monomer and homo-octamer structures. Initially, 1,523 contig sequences were retrieved from the RNA Deep Virome Assemblage (RDVA) database (Edgar et al., 2022; Forgia et al., 2023) by tblastn (*E*-value threshold: 1.00 × 10^−10^). Subsequently, corresponding open reading frames (ORFs) were extracted and translated. After filtering identical sequences, 752 polypeptide sequences homologous to AfViV1-CP were obtained. A custom MSA generated by Clustal Omega was used as an input for AlphaFold2 structural prediction using locally-installed ColabFold version 1.5.1 [https://github.com/YoshitakaMo/localcolabfold; (Evans et al., 2021; Jumper et al., 2021; Mirdita et al., 2022)]. The alphafold2_ptm (with 3 recycles) and alphafold2_multimer_v3 (with 20 recycles) model types were used for monomer and multimer predictions, respectively. Superimposition of models by the Matchmaker tool and visualization were performed using ChimeraX software version (v1.9) (Pettersen et al., 2021). The high confidence region of the monomer model [mostly predicted local distance difference test (pLDDT) > 80, amino acids (aa) 50–221] was used as an input for a Foldseek search on the webserver (https://search.foldseek.com) (Van Kempen et al., 2024).

### 2.5 qRT-PCR

Total RNA was extracted from each fraction using TRIzol and a PureLink kit (Thermo Fisher Scientific) according to the manufacturer’s protocol. An equal amount of RNA solution was applied during cDNA synthesis and qPCR using an iTaq Universal SYBR Green One-Step kit (Bio-Rad, USA) and CFX Connect real-time PCR detection system (Bio-Rad, CA, USA) Reverse transcription was performed at 50°C for 10 min, followed by 1 min at 95°C to activate DNA polymerase; for PCR, 40 cycles of denaturation was conducted at 95°C for 10 s, followed by annealing and extension at 60°C for 30 s. Melting curve analysis was performed following qRT-PCR to confirm the specificity of the amplified products. The forward and reverse primers were listed in N.

### 2.6 Phylogenetic analysis

Phylogenetic analyses of the RdRp of AfViV1 and related viruses involved retrieving amino acid sequences from the National Center for Biotechnology Information non-redundant (NCBI nr) database. AfViV1-related sequences were selected based on the results of a BLASTp search against the NCBI nr database, and only sequences with an E-value of 0 were included. Similarly, Aspergillus flavus virga-like virus 1, which is also predicted to be classified as a vivivirus, was analyzed using a BLASTp search, and only sequences with an E-value less than 1e-10 were utilized for the analysis. The sequences were alignment using MUSCLE (Edgar, 2004) within MEGA11 (Tamura et al., 2021). Ambiguously aligned positions were excluded with trimAl (option: -gt 0.8) (Capella-Gutiérrez et al., 2009). Maximum likelihood phylogenetic analyses were performed using RAxML (Stamatakis, 2014), with bootstrap tests conducted using 1,000 replicates. The amino acid substitution model was selected by ProtTest (Abascal et al., 2005) based on Akaike’s information criterion (Sugiura, 1978). Phylogenetic trees were prepared based on the maximum likelihood (ML) and neighbor-joining (NJ) methods and visualized using MEGA11.

## 3 Results

### 3.1 Phylogenetic position of AfViV1

**Fig. 1:**
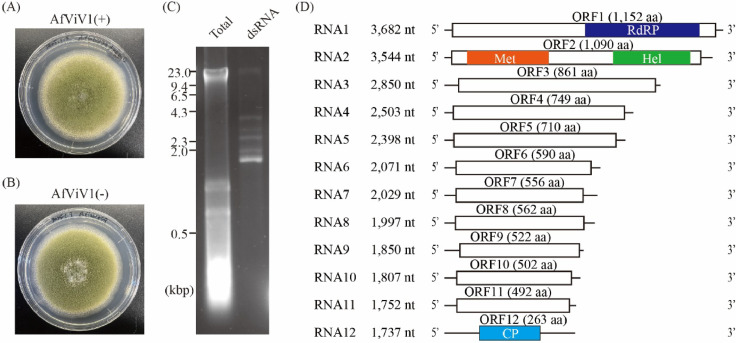
Characteristics of AfViV1. Colony morphology of AfViV1-infected *A. flavus* (A) or the AfViV1-cured strain (B) on PDA. (C) Electrophoretic pattern of total nucleic acids and dsRNA extracted from AfViV1-infected *A. flavus*. (D) The RNA genome structure model for AfViV1. White boxes indicate the predicted ORFs. The domains identified as methyltransferase, RdRP, and helicase are highlighted in orange, dark blue, and green boxes, respectively. The CP highlighted in blue was determined from purified virus particles in this study.

**Fig. 2:**
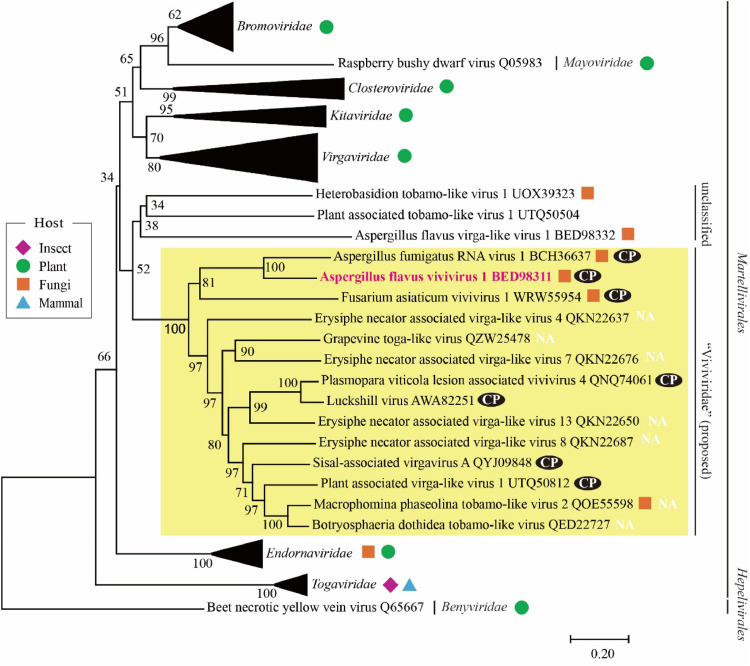
Phylogenetic analysis of RdRps found in AfViV1 and related RNA viruses using the NJ method. The number above each branch indicates the bootstrap values, with only those greater than 50% displayed. The scale bars represent substitutions per site, and the yellow area highlights the clade emphasized in this study. The CP icon indicates that AfViV1-CP related amino acid sequences are detected from the virus or corresponding SRA data. NA indicates that the SRA data could not be accessed.

AfViV1 had been identified from *A. flavus* IFM61879 (Fig. 1). AfViV1 did not induce symptoms (Fig. 1A and B). The AfViV1-infected strain showed several dsRNA bands, and sequencing analysis of the dsRNA revealed twelve RNA segments (Fig. 1C and D). Viviviruses infecting fungi form a sister clade with *Virgaviridae* (Chiapello et al., 2020). Meanwhile, another report suggests that viviviruses form a clade next to *Togaviridae* (Medd et al., 2018). To confirm the phylogenetic position of AfViV1 and related viruses, phylogenetic analysis was conducted using RdRP sequences belonging to the *Martellivirales* order. The AfViV1-related sequences were corrected based on the Blastp search against the NCBI nr database (see Materials and Methods).. Our NJ analysis showed that AfViV1 and related viviviruses formed a single clade, which was positioned between plant viruses (*Bromoviridae, Mayoviridae, Closteroviridae, Kitaviridae*, and *Virgaviridae* families) and viruses in the *Endornaviridae* family infecting plants and fungi (Fig. 2). In this analysis, viruses in the *Togaviridae* family infecting mammals and insects were the first to diverge in the order *Martellivirales*. AfViV1 and related viviviruses formed a sister clade with *Togaviridae* using the ML method, although the bootstrap support was below 50 (Fig. S1). In this case, plant viruses also formed a single clade. These results suggest that fungal viruses, including AfViV1 and related viviviruses, may form a single group in the order *Martellivirales*. The relationship to the previously established families is unclear, although it seems to reflect the phylogenetic differences of their hosts (Fig. 2 and S1).

### 3.2 Virus particles of AfViV1

**Fig. 3:**
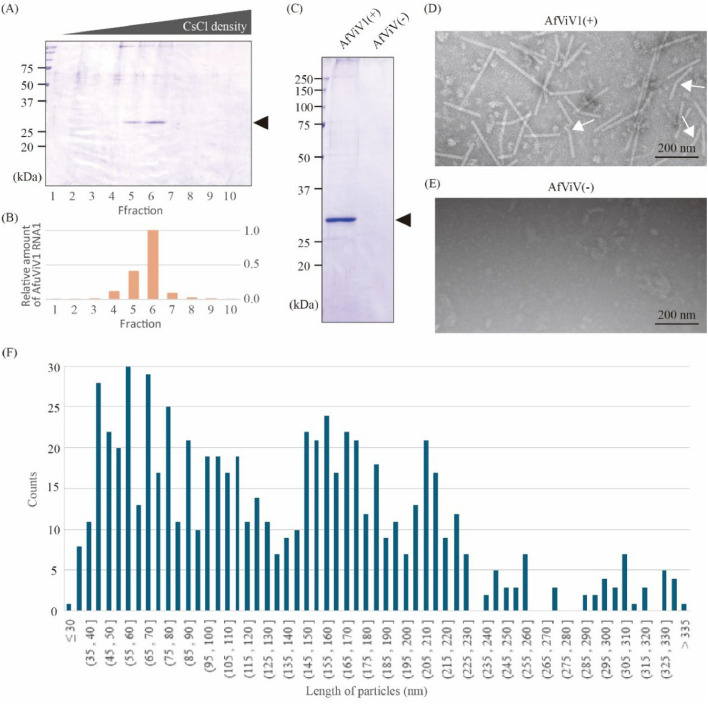
Purification and characterization of AfViV1 virus particles. (A) SDS-PAGE profile of CsCl fractions. The arrowhead indicates the CP band. (B) qRT-PCR showing the relative amount of AfViV1 RNA1 in each fraction. (C) SDS-PAGE profile of the purified virus particle fraction obtained from AfViV1-infected *A. flavus* [AfViV1(+)] and the AfViV1-cured strain [AfViV1(-)]. The arrowhead indicates the CP band. Negative-contrast electron micrograph of the purified virus particle fraction obtained from AfViV1-infected *A. flavus* (D) and the AfViV1-cured strain (E). (F) Length distribution of AfViV1 particles.

Based on the close relationship of AfViV1 to plant viruses (see above), we applied a standard sucrose gradient method for plant virus purification (Urayama et al., 2010; Yue et al., 2024). The AfViV1 particle was mostly detected in fraction 4 according to the detection of a 29 kDa major protein band by SDS-PAGE analysis (Fig. S2A). Similarly, AfViV1 RNA1 accumulated in fraction 4 according to qRT-PCR analysis (Fig. S2B). Further separation of fraction 4 using a CsCl density gradient, followed by SDS-PAGE and qRT-PCR analysis suggests that AfViV1 particles are composed of a 29 kDa CP and have a density of 1.265 g.cm^−3^ (Fig. 3A–C and Fig. S3). Nano LC-MS/MS analysis showed that the 29 kDa band corresponded to ORF12 of AfViV1 (calculated molecular mass: 29.2 kDa) (Fig. 1D). This purified fraction contains rod-shaped particles that were not detected in the AfViV1-cured strain sample (Fig. 3D and E). The rod-shaped particles exhibited various lengths (Fig. 3F), suggesting that each particle contains a single RNA molecule. Additionally, a minor fraction of particles were kinked or curved (Fig. 3D, white arrow).

### 3.3 Identification of the CP of other vivivirues

**Fig. 4:**
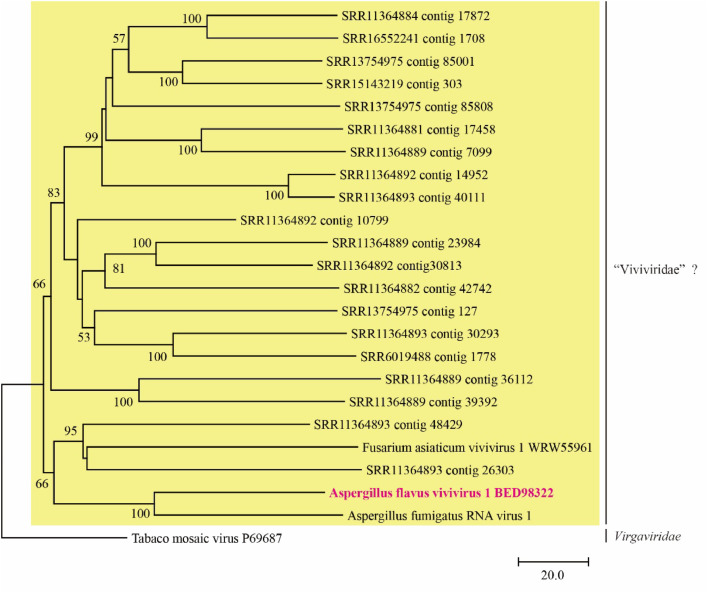
Phylogenetic analysis of CPs found in AfViV1 and related RNA viruses using the NJ method. The number above each branch indicates the bootstrap values, with only those greater than 50% displayed. The scale bars represent substitutions per site, and the yellow area highlights the clade emphasized in this study. Three sequences that exhibited over 99% amino acid sequence identity to SRR11364892_contig_10799 were excluded: SRR11364878_contig_1157, SRR11364880_contig_22816, and SRR11364885_contig_17291.

To demonstrate the conservation of CP in the viruses within this clade, we obtained sequence read archive (SRA) data containing AfViV1-related RdRPs identified through a Blastp search against the NCBI nr database (*E*-value = 0.0). The trimmed short reads were assembled and the resulting contigs were used as queries for a Blastx search against AfViV1-CP and AfViV1-RdRP. Consequently, many SRAs yielded sequences related to AfViV1-RdRP and AfViV1-CP (*E*-value ≤ 1 × 10^−10^), while some SRAs only yielded RdRP sequences (Table 1). Notably, RdRP was always detected in SRAs where CP was identified. To confirm the correlation of CP with RdRP, we also downloaded several SRA data, including AfViV1-CP related sequences found using a tblastn search against the RDVA of the Serratus platform (Edgar et al., 2022; Forgia et al., 2023). In this case, all SRA included RdRPs related to AfViV1 (Table 1). These data show that AfViV1-CP-related CPs are common in this “Viviviridae” clade (Fig. 2), although they were not identified in previous reports. Importantly, the SRR11364878–SRR11364893 data used for this analysis was identical to the SRA dataset used in the original paper proposing “Viviviridae” (Chiapello et al., 2020). The CP-based phylogeny indicates that “Viviviridae” form a distinct clade from viruses in the *Virgaviridae* family (Figs. 4 and S4).

**Table 1:** Co-occurrence of AfViV1-RdRP and AfViV1-CP-related sequences in metatranscriptome SRAs.

SRA accession	Source*	RdRP	CP	Sample
SRR10946722	Blast_RdRP	✓	✓	soil microbial communities
SRR16552241	Blast_RdRP	✓	✓	symptomatic (virus disease-like) tomato plants
SRR6019488	Blast_RdRP	✓	✓	wild-caught *Drosophila suzukii*
SRR13754975	Blast_RdRP	✓	✓	plant
SRR13754976	Blast_RdRP	✓	✓	
SRR11364878	Blast_RdRP	✓	✓	grapevine downy mildew lesions
SRR11364879	Blast_RdRP	✓	✓	
SRR11364880	Blast_RdRP	✓	✓	
SRR11364881	Blast_RdRP	✓	✓	
SRR11364882	Blast_RdRP	✓	✓	
SRR11364883	Blast_RdRP	✓	✓	
SRR11364884	Blast_RdRP	✓		
SRR11364885	Blast_RdRP	✓	✓	
SRR11364886	Blast_RdRP	✓	✓	
SRR11364887	Blast_RdRP	✓		
SRR11364888	Blast_RdRP	✓		
SRR11364889	Blast_RdRP	✓	✓	
SRR11364890	Blast_RdRP	✓	✓	
SRR11364891	Blast_RdRP	✓		
SRR11364892	Blast_RdRP	✓	✓	
SRR11364893	Blast_RdRP	✓	✓	
SRR15143225	Blast_RdRP	✓	✓	sisal
SRR15143226	Blast_RdRP	✓	✓	
SRR15143227	Blast_RdRP	✓		
SRR15143235	Blast_RdRP	✓	✓	
SRR15143236	Blast_RdRP	✓	✓	
SRR15143239	Blast_RdRP	✓		
SRR15143240	Blast_RdRP	✓		
SRR15143219	Blast_RdRP	✓	✓	
SRR15143220	Blast_RdRP	✓	✓	
SRR15143221	Blast_RdRP	✓		
DRR089003	RDVA_CP	✓	✓	fermented grains
DRR089004	RDVA_CP	✓	✓	
SRR2063931	RDVA_CP	✓	✓	bat virome
SRR5756505	RDVA_CP	✓	✓	plant sample from *Litchi chinensis* mycorrhizal root
SRR10919503	RDVA_CP	✓	✓	soil microbial communities
SRR10920923	RDVA_CP	✓	✓	
SRR10933884	RDVA_CP	✓	✓	

⁎ : The SRA was selected based on the Blast search result against the NCBI nr database using AfViV1-RdRP as the query (Blast_RdRP) or the tblastn search result against the RDVA database using AfViV1-CP as the query (RDVA_CP)

### 3.4 Predicted structural similarity between AfViV1-CP and PVY-CP

**Fig. 5:**
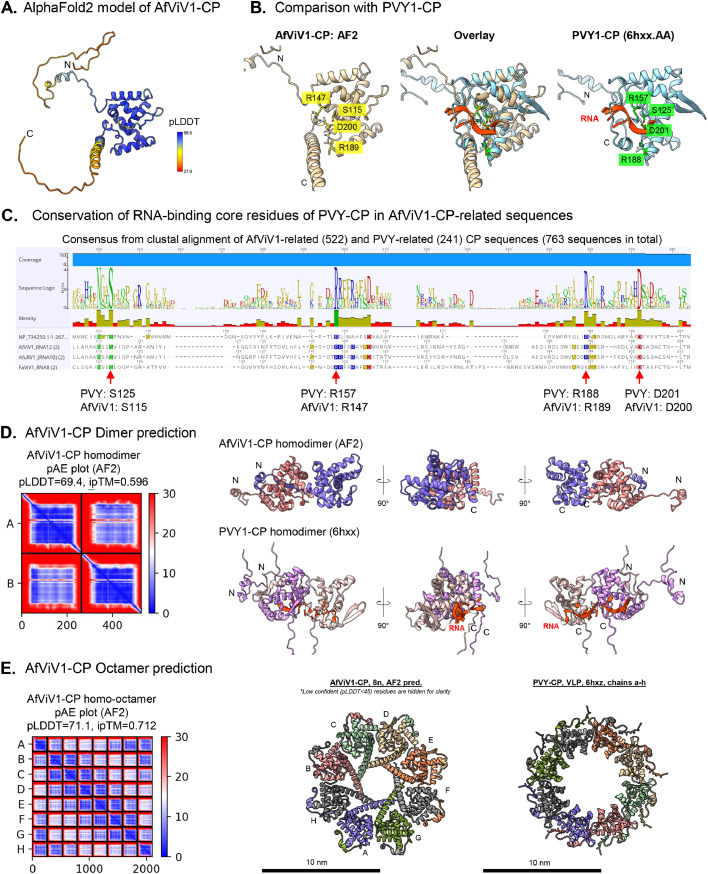
AfViV1-CP monomer and multimer structural prediction. (A) AlphaFold2 (AF2) prediction of AfViV1-CP monomer [predicted local distance difference test (pLDDT) = 76.3, predicted template modeling (pTM) = 0.657]. (B) Comparison and structural alignment of the AfViV1-CP AF2 model against PVY1-CP [PDB: 6HXX, chain AA (Kežar et al., 2019)]. The core RNA binding residues in PVY-CP and their corresponding residues in AfViV1-CP are highlighted. (C) Conservation of PVY-CP RNA-binding residues among AfViV1-CP. Clustal Omega amino acid sequence alignment of 522 AfViV1-CP homologs and PVY-CP homologs analyzed using Geneious Prime. (D) AfViV1-CP homodimer prediction and comparison with the PVY-CP dimer. A predicted aligned error (pAE) plot, confidence scores (pLDDT/interface predicted template modelling [ipTM]) for the multimer prediction, and side-by-side comparison of homodimer models for AfViV1-CP (AF2) and PVY-CP (PDB: 6HXX). (E) AfViV1-CP homo-octamer prediction and comparison with PVY-CP virus-like particle [VLP, PDB: 6HXZ (Kavčič et al., 2024)]. The color keys in (D) and (E) show the pAE in Å.

To gain further insights into the characteristics of the AfViV1-CP, we used AlphaFold2 to predict the monomer and multimer structures (Fig. 5). To enable robust prediction, we generated a custom MSA using 752 AfViV1-CP homologous sequences from the RDVA database obtained by tblastn (*E*-value threshold: 1.00 × 10^−10^, see Methods). The predicted AfViV1-CP structure showed a well-folded globular domain flanked by low-confidence N- and C-termini (Fig. 5A). Subsequently, we looked for structurally similar proteins in the Big Fantastic Virus Database (BFVD) and in the Protein Data Bank (PDB) using Foldseek (Berman et al., 2000; Kim et al., 2025; Van Kempen et al., 2024). With the high-confidence region of the AfViV1-CP predicted model as a query (aa 50–221), the highest hit was an AlphaFold2-predicted structure of Carrot yellow leaf virus CP (*Closteroviridae*, A0A0A0P5L9; probability, 1.00; sequence identity, 15.8%; *E*-value, 1.42 × 10^−3^; score, 153), along with those from *Closteroviridae* and *Potyviridae* filamentous viruses according to the BFVD search (Fig. S5, Table S2). These structural homologies among the predicted models are consistent with the observed filamentous nature of AfViV1 virus particles. Furthermore, Potato Virus Y viral particle was the highest probability match among experimentally determined structures in the PDB (PVY-CP, PDB-6HXX; probability, 0.998; sequence identity, 12.7%; *E*-value, 1.48 × 10^−1^; score, 93). Despite the large difference in overall amino acid sequences (6.53% identity), the AfViV1-CP structural prediction exhibited characteristics of the PVY-CP structure including a globular core subdomain with seven to eight α-helices, and a 13-aa loop with a short beta-hairpin in place of a 14-aa beta-hairpin in the PVY-CP (Fig. 5B).

Structural alignment of AfViV1-CP with the high-resolution cryo-electron microscopy (EM) structure of PVY-CP (Kežar et al., 2019) suggests that AfViV1-CP has the same core RNA-binding residues as PVY-CP in their respective positions (Fig. 5B, highlighted residues). If the potential core RNA-binding residues of AfViV1-CP are functionally important, these residues would be conserved among AfViV1-like sequences. Sequence alignment of 522 RDVA-derived AfViV1-CP homologous sequences (>150-aa) and 241 PVY-CP homologous sequences (obtained by Blastp, Refseq) showed that the four core RNA-binding residues of PVY-CP were almost perfectly conserved in the AfViV1-CP, despite the overall sequence divergence between the two groups (Fig. 5C). This indicates that AfViV1-CP may bind RNA in a manner analogous to that observed for PVY-CP, conserving the same set of core RNA-binding residues.

The PVY viral particle is a long flexuous filament (Kavčič et al., 2024; Zamora et al., 2017). To test whether AfViV1-CP could form homo-oligomers like PVY-CP, we modeled its dimer and homo-octamer models (Fig. 5D, E). The AfViV1 homodimer model exhibited a flexible N-terminal region reaching and interacting with the adjacent unit, as seen in the PVY-CP N-terminal region (Fig. 5D). The homo-octamer model showed a unit distribution pattern like the RNA-free virus-like particle of PVY-CP, and the C-terminal helix regions positioned in the interior of the octamer ring structure (Fig. 5E). This indicates that AfViV1-CP may oligomerize in a similar fashion to that of PVY-CP.

### 3.5 Re-analysis of AfuRV1, the virus most closely related to AfViV1

The top hit sequence of AfViV1-RdRP was Aspergillus fumigatus RNA virus 1 (AfuRV1) according to fragmented and primer-ligated dsRNA sequencing (FLDS) technology (Chiba et al., 2021). The amino acid identity of their RdRPs was 60%, suggesting that these viruses share some features, including particle and genome structures. However, AfuRV1 has only three RNA segments, and the CP gene was not recognized (Chiba et al., 2021). Therefore, we prepared a new FLDS library and re-analyzed the new (DRR639660 and DRR639661) and previously reported (DRR235409) FLDS raw data. Finally, we identified additional RNA segments that were overlooked in previous analyses (Fig. S6). AfuRV1 contains 10 RNA segments, and ORF10 of RNA10 shows significant similarity to AfViV1-CP (53.0% sequence identity).

## 4 Discussion

Our data show that AfViV1 exhibits rod-shaped particles. Notably, rod-shaped particles of varying lengths have not been previously reported in fungal viruses in the *Kitrinoviricota* phylum, including the *Martellivirales* order. Additionally, we identified CP and found that it represents a rare case wherein it is encoded in a relatively short segment. Furthermore, our analysis of publicly available next-generation sequencing (NGS) data suggests that these characteristics are likely shared by members of the proposed group, Viviviridae, which appears to represent this group as a single viral family. Our analysis of this novel RNA virus clade expands the current understanding of the diversity in morphology and genome structure among fungal RNA viruses. In a previous study (Degola et al., 2021), purification of virus particles from a strain co-infected with AfViV1 and Aspergillus flavus deltaflexivirus 1 revealed two distinct structures: filamentous structures and non-enveloped isometric particles. Recent studies have further suggested that deltaflexi-related viruses have isometric virus particles (Wu et al., 2024). These findings strongly indicate that AfViV1 is associated with rod-shaped particles (“filamentous” in the original article). Furthermore, Jiatao Xie’s group recently characterized Fusarium asiaticum vivivirus 1 (FaVvV1) to have rod-shaped virions, and suggested a potential evolutionary relationship between FaVvV1 and plant-infecting potyviruses (Huang et al., 2025)

In addition, many fungal and plant viruses are taxonomically related and known to be host-compatible (Andika et al., 2023; Telengech et al., 2024). This study, which strengthens the unexpected similarity between fungal and plant viruses, raises the possibility that there may be additional inter-kingdom transmission pathways for fungal and plant viruses. Such potential transmission routes may involve ecological interfaces where fungi and plants closely interact, such as in the rhizosphere or during endophytic colonization (Andika et al., 2023). Given that certain viruses can replicate in both fungal and plant hosts, these shared niches could serve as bridges facilitating viral movement across kingdom boundaries. The discovery of mycoviruses with sequence similarities to plant viruses further supports this hypothesis. Further investigations into these ecological overlaps may provide important clues about the mechanisms enabling cross-kingdom infections.

In our analysis, the phylogenetic position of the Viviviridae clade (including AfViV1, based on the RdRP sequence) remains unclear. Furthermore, the amino acid sequence and predicted structural characteristics of the CP showed considerable divergence from those of the *Virgaviridae* and *Togaviridae* families, precluding any definitive conclusions regarding their relatedness. However, the rod-shaped particles of AfViV1 appeared more similar to viruses in the *Virgaviridae* family that possess rod-shaped particles compared with viruses in the *Togaviridae* family that exhibit spherical particles. Upon reviewing the several phylogenetic trees reported for these viviviruses, most did not encompass all families within the *Martellivirales* order. Consequently, it remains unclear whether the analyzed RdRP sequences are closest to the *Virgaviridae* family, or if their placement simply reflects the exclusion of certain families from the analysis. One possible reason is the difficulty in constructing a reliable phylogenetic tree that includes all families within the *Martellivirales* order. Although our phylogenetic tree covers all families within this order, it lacks sufficient bootstrap support. Understanding the origin and evolutionary pathways of Viviviridae requires completing gaps in RNA virus diversity.

In the NJ tree of AfViV1 RdRP, viviviruses show the closest affinity to Aspergillus flavus virga-like virus 1 and a group of mycoviruses referred to as the tobamo-like virus clade. The ML tree analysis, after excluding poorly supported branches (such as Endornaviridae and Togaviridae with low bootstrap values), similarly demonstrates a close relationship between viviviruses, A. flavus virga-like virus 1, and the tobamo-like virus clade. Furthermore, a recent study (Hong et al., 2025) reported that Nigrospora aurantiaca tobamo-like virus 1 (NaTLV1) forms filamentous particles, and its CP is more similar to those of Betaflexiviridae/Gammaflexiviridae than to those of Virgaviridae. These findings suggest that viviviruses share similarities with tobamo-like viruses in both RdRP and CP characteristics.

AfViV1 particles exhibit a linear morphology reminiscent of rods, but based on diameter, pitch (particle length/RNA base), predicted CP structure, and kinked subpopulation, the characteristics were similar to filamentous virus particles. The 14–16 nm diameter of AfViV1 particles was thinner than rod-shaped TMV (*Virgaviridae*) and SsNSRV-1 (*Mymonaviridae*) (20 nm), and closer to that of filamentous PVY (*Potyviridae*) and BYV (*Closteroviridae*) (10–15 nm). The longest population of AfViV1 particles was approximately 330 nm, and may correspond to AfViV1 RNA1 (3,682 nt). The calculated pitch was 89 nm.kb^−1^. This was higher than rod-shaped TMV (approximately 47 nm.kb^−1^) and SsNSRV-1 (approximately 35 nm.kb^−1^), and closer to that of filamentous PVY (approximately 85 nm.kb^−1^) and BYV (approximately 84 nm.kb^−1^). These observations are consistent with the predicted CP-CP and CP-RNA interactions in AfViV1 particles that resemble those of potyvirus and closterovirus. The structural similarity between AfViV1-CP and PVY-CP was unexpected, given their extremely low amino acid sequence identity and the distant relationship inferred from the RdRP-based phylogeny. However, similar phenomena are reported among CPs of phylogenetically distant viruses (Zamora et al., 2017). For instance, the CP fold of the plant (+)ssRNA virus, Pepino mosaic virus, resembles that of nucleoproteins from animal (-)ssRNA viruses (Agirrezabala et al., 2015). This study suggests that differences in C-terminal extensions of the CP may determine the characteristics of distinct multimeric assemblies (a flexuous, helical rod or a loose ribonucleoprotein). The difference in particle shape between AfViV1 (rod-shaped) and PVY (flexuous filament) might originate from subtle structural differences. The potyvirus CP protein fold and RNA binding site is conserved among other eukaryotic viruses that have distinct virion shapes, including icosahedral particles of Rift Valley fever virus (*Phlebovirus* in the *Bunyaviridae* family) (Zamora et al., 2017). High-resolution reconstruction of the AfViV1 viral particle using cryo-EM would provide further insights to explain how AfViV1-CP contributes to its rod-shaped virion architecture.

## Funding information

This research was supported by a grant from the Institute for Fermentation, Osaka, and in part by a Grant-in-Aid for Scientific Research (21K18217 and 24K02083) from the Ministry of Education, Culture, Sports, Science, and Technology (MEXT) of Japan, and by Lilly Endowment, Inc., through its support for the Indiana University Pervasive Technology Institute which provided supercomputing resources for protein structure modelling.

## CRediT authorship contribution statement

**Mika Yoshioka:** Writing – original draft, Visualization, Formal analysis. **Akihito Fukudome:** Writing – review & editing, Writing – original draft, Visualization, Formal analysis, Data curation. **Yuto Chiba:** Writing – review & editing, Data curation. **Daisuke Hagiwara:** Writing – review & editing, Funding acquisition, Conceptualization. **Syun-ichi Urayama:** Writing – review & editing, Writing – original draft, Visualization, Funding acquisition, Formal analysis, Conceptualization.

## Declaration of competing interest

The authors declare that they have no known competing financial interests or personal relationships that could have appeared to influence the work reported in this paper.

## Data Availability

Data will be made available on request.

## Appendix Supplementary materials

Image, application 1

Image, application 2
