# Supplementary material for: Characterization of a multi-segmented rod-shaped mycovirus within the order Martellivirales largely accommodating plant viruses
Source: Virus Res. 2025 May 30;357:199591. doi: 10.1016/j.virusres.2025.199591 (PMC12169780; doi:10.1016/j.virusres.2025.199591)
Supplement: Supplementary file 1 [file mmc1.docx]

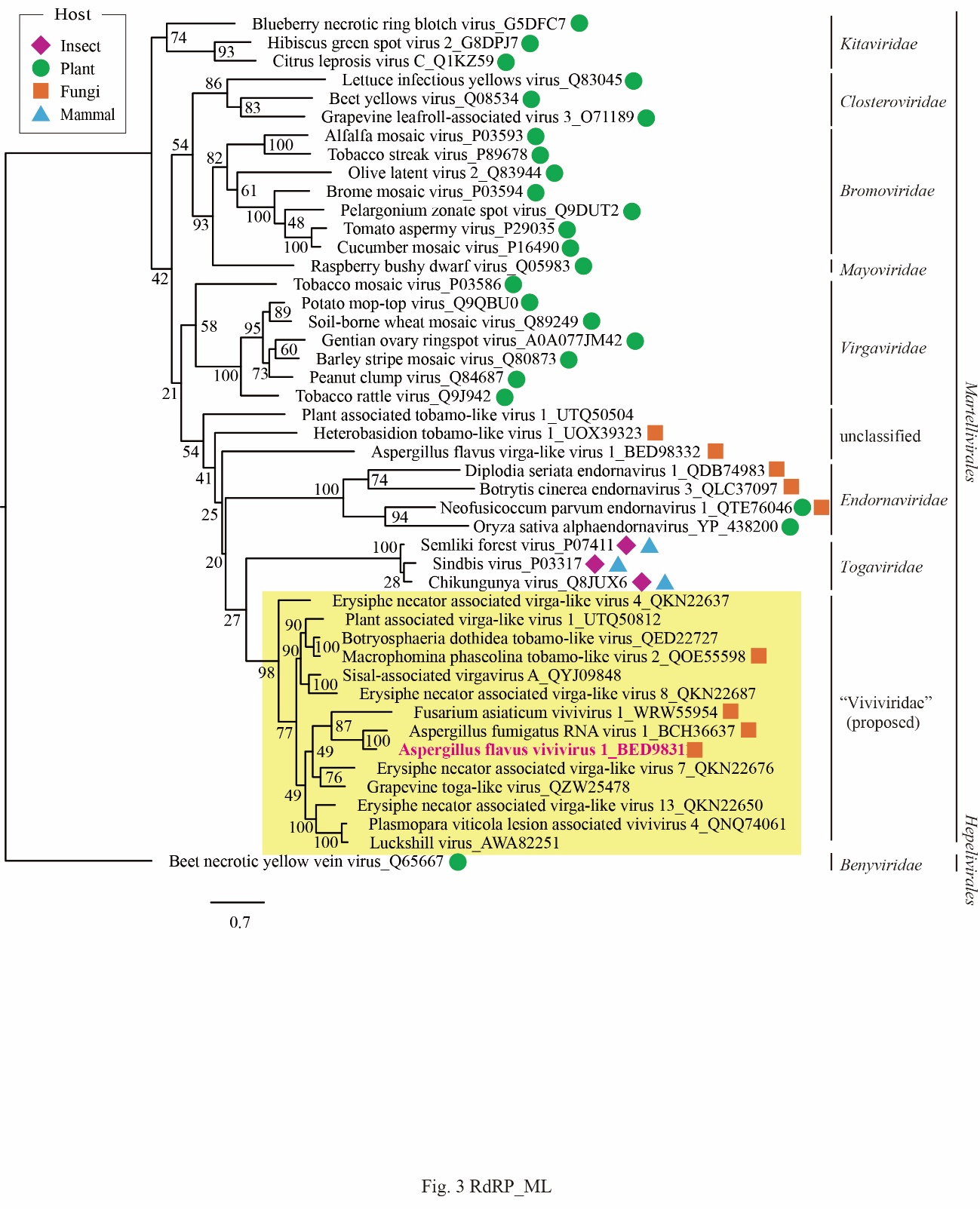


Fig. S1: Phylogenetic analysis of RdRps found in AfViV1 and related RNA viruses using the ML method

The number above each branch indicates the bootstrap values, with only those greater than 50% displayed. RAxML was used with the LG+I+G+F model. The scale bars represent substitutions per site, and the yellow area highlights the clade emphasized in this study.


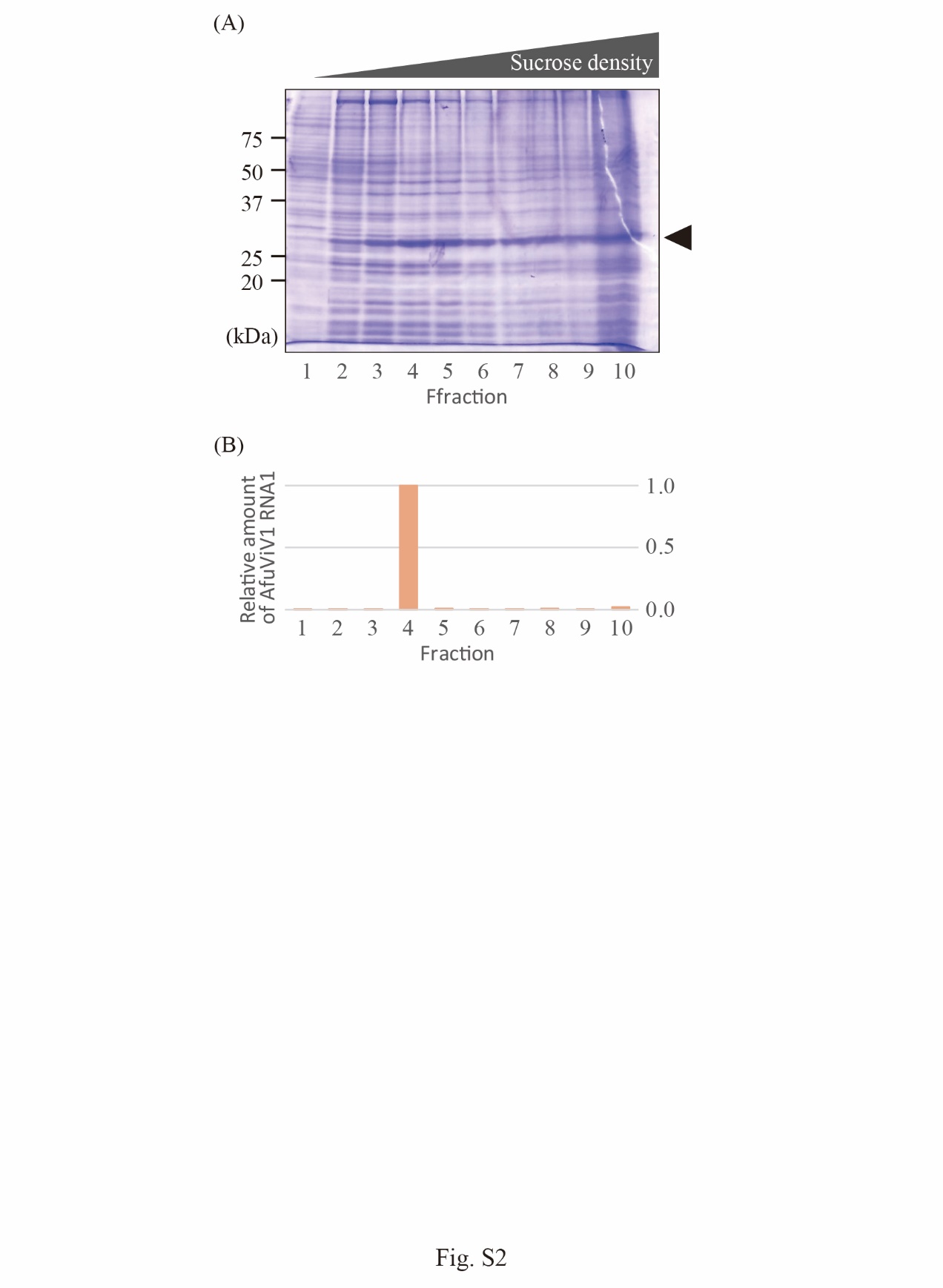


Fig. S2: Purification of AfViV1 virus particles using a sucrose gradient

(A) SDS-PAGE profile of sucrose gradient fractions. The arrowhead indicates the position of the CP band. (B) qRT-PCR showing the relative amount of AfViV1 RNA1 in each fraction.


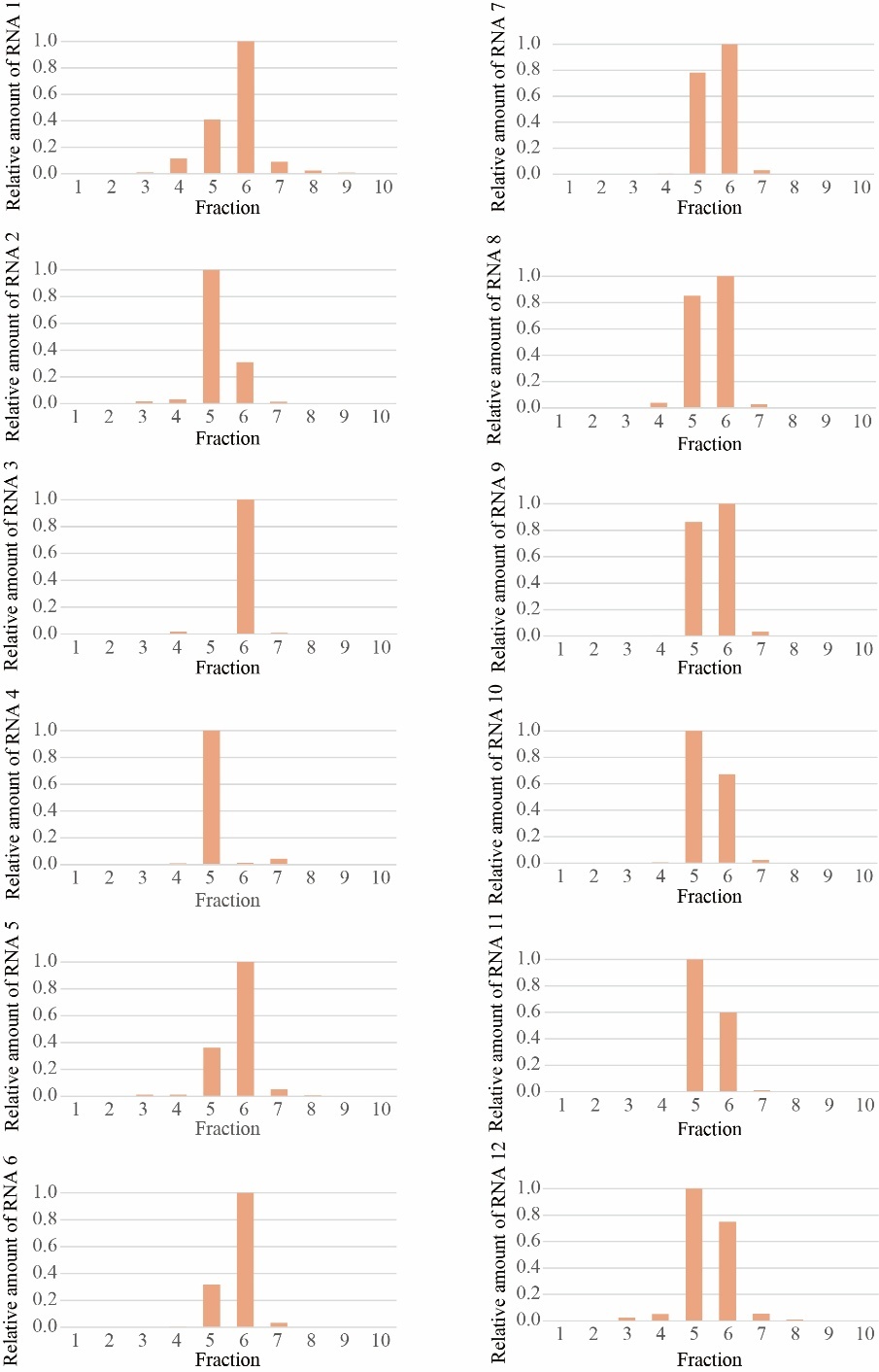


Fig. S3: Relative abundance of AfViV1 RNAs in each fraction.

qRT-PCR was used to assess the distribution of AfViV1 RNAs among the fractionated samples.


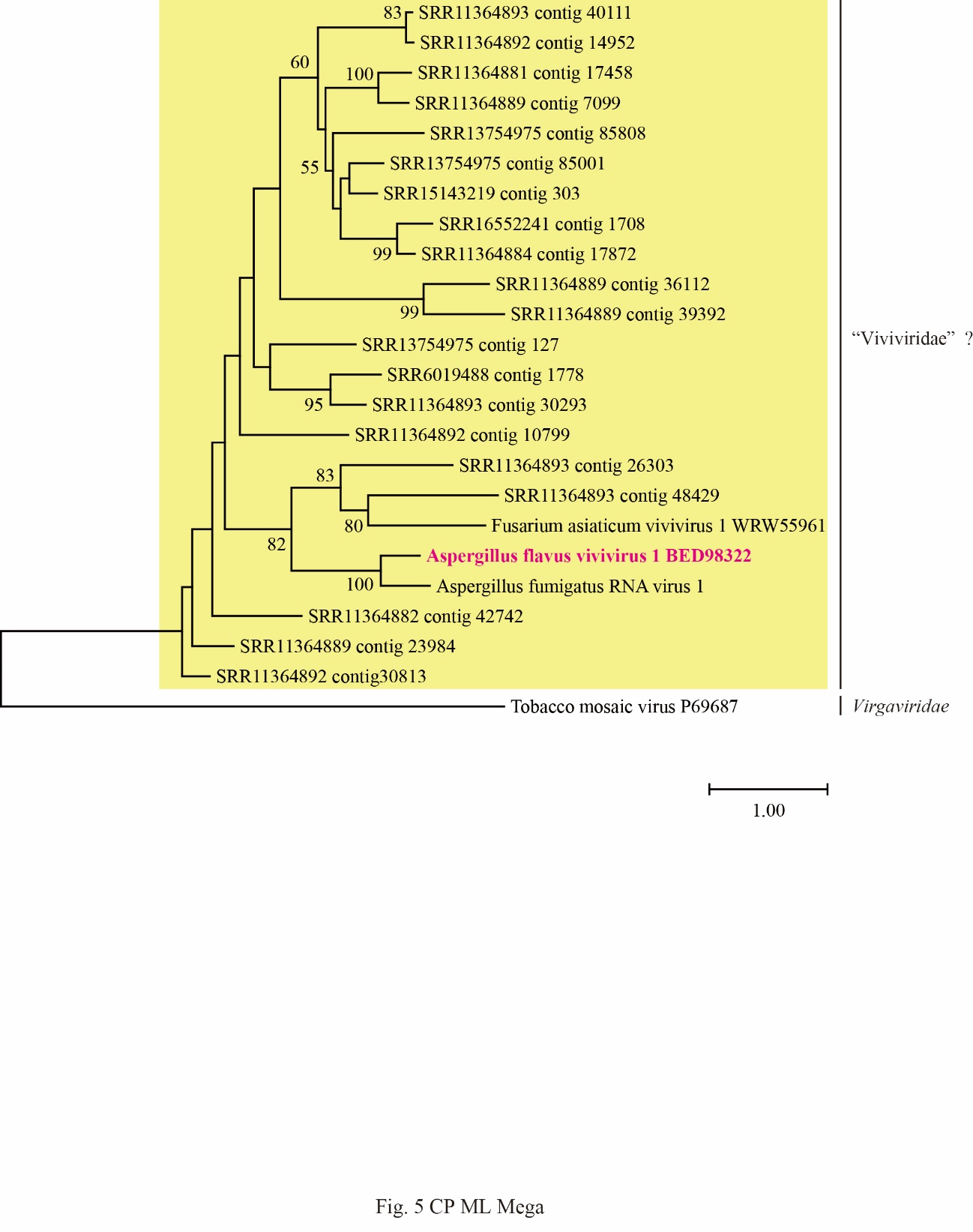


Fig. S4: Phylogenetic analysis of CP found in AfViV1 and related RNA viruses using the ML method

The number above each branch indicates the bootstrap values, with only those greater than 50% displayed. RAxML was used with the LG+I+G+F model. The scale bars represent substitutions per site, and the yellow area highlights the clade emphasized in this study. Three sequences that exhibited over 99% amino acid sequence identity to SRR11364892_contig_10799 were excluded: SRR11364878_contig_1157, SRR11364880_contig_22816, and SRR11364885_contig_17291.

Fig. S5: AfViV1-CP Foldseek hit protein models


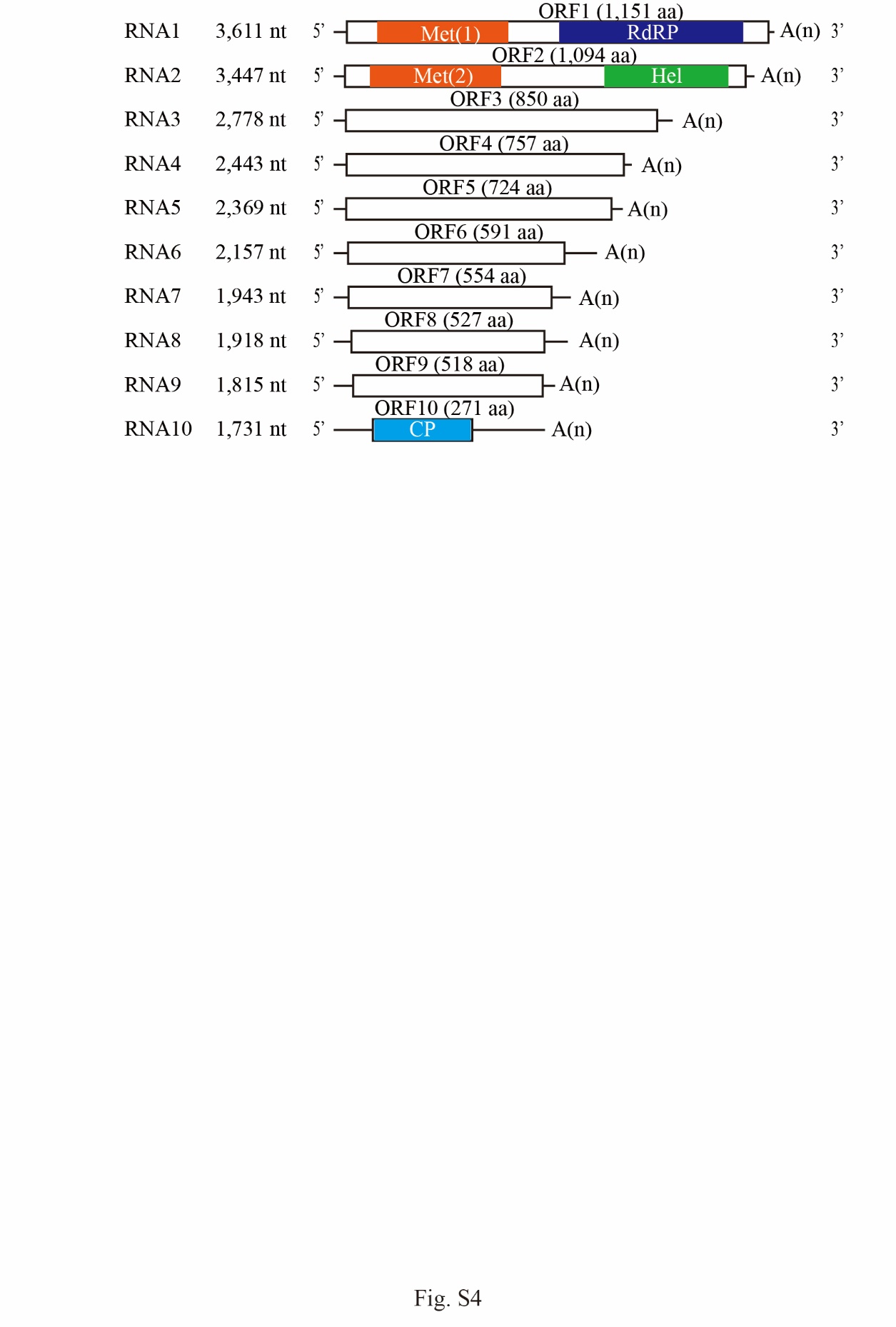


Fig. S6: Updated AfuRV1 genome

The RNA genome structure model for AfuRV1. White boxes indicate the predicted ORFs. The domains identified as methyltransferase, RdRP, and helicase are indicated in orange, dark blue, and green boxes, respectively. The CP in light blue was determined using the AfViV1-CP sequence.

Table S1: Primers used for qRT-PCR analysis

| Primer name | Sequence 5' -> 3' | Target |
| --- | --- | --- |
| AfViV1_RNA1_F2 | CTGGTGTGTAAGCGGATGGT | AfViV1 RNA1 |
| AfViV1_RNA1_R2 | CAACAACGCCTCCCTCTGAT |  |
| AfViV1_RNA2_F1 | TGTCTACGATGTGCCTGTGG | AfViV1 RNA2 |
| AfViV1_RNA2_R1 | GCCTGACCCTACAAAACCGA |  |
| AfViV1_RNA3_F1 | GAGGTAGTGCGGAAAGAGGC | AfViV1 RNA3 |
| AfViV1_RNA3_R1 | GCGATAAATGCCCAACAGCG |  |
| AfViV1_RNA4_F1 | ACGAGAAGGCAAAGGCACG | AfViV1 RNA4 |
| AfViV1_RNA4_R1 | GCGGTCAAAGTAGCAAAGGC |  |
| AfViV1_RNA5_F1 | AGGCTAACGGTGTGGGTTTG | AfViV1 RNA5 |
| AfViV1_RNA5_R1 | ACCTGCGTCTTACTACTGCG |  |
| AfViV1_RNA6_F1 | TCACGACGAGTGCTATGAGG | AfViV1 RNA6 |
| AfViV1_RNA6_R1 | AGACACACAGGTTCGGTTCC |  |
| AfViV1_RNA7_F1 | GAGAGGGCAACGGCTTATCA | AfViV1 RNA7 |
| AfViV1_RNA7_R1 | GCTCGCATCACCACTTTACC |  |
| AfViV1_RNA8_F1 | TGAACGGTGTTGCTGGATGG | AfViV1 RNA8 |
| AfViV1_RNA8_R1 | GCTCAGGCTCACTGCTAACC |  |
| AfViV1_RNA9_F1 | CGATTGTCCACGCCATTTGT | AfViV1 RNA9 |
| AfViV1_RNA9_R1 | CAGTAGCCGTCCTCCTTGTG |  |
| AfViV1_RNA10_F1 | GATTGTCGGTGTGGGCAAAG | AfViV1 RNA10 |
| AfViV1_RNA10_R1 | CTACCTCTCTCGTCGTTCGG |  |
| AfViV1_RNA11_F1 | TCTACCGCCCGAAATGACTG | AfViV1 RNA11 |
| AfViV1_RNA11_R1 | GAAAACCCGCAACTACCACG |  |
| AfViV1_RNA12_F1 | CTTCTTGGGCGACGATTCAC | AfViV1 RNA12 |
| AfViV1_RNA12_R1 | TCGGCACTATCAGCAACCAG |  |

Table S2: AfViV1-CP Foldseek search BFVD and PDB hits

(see Table S2.xlsx)
